# Depression and chronic kidney disease: a cross-sectional study based at Bugando Medical Centre, Northwestern Tanzania

**DOI:** 10.11604/pamj.2022.42.297.31414

**Published:** 2022-08-22

**Authors:** Hulda Munisi, Irene Sylvester, Isaya Mwakalebela, Said Kanenda, Ladius Rudovick, Matiko Mwita, Elias Nyanza

**Affiliations:** 1 Catholic University of Health and Allied Sciences, Bugando, Mwanza, Tanzania,; 2 Bugando Medical Centre (BMC), Nephrology Unit, Bugando, Mwanza, Tanzania,; 3 Bugando Medical Centre (BMC), Psychiatry Department, Bugando, Mwanza, Tanzania,

**Keywords:** Chronic Kidney diseases, depression, dialysis, end-stage renal disease

## Abstract

**Introduction:**

psychosocial distress such as depression is prevalent among chronic kidney disease (CKD) patients. It is often overlooked despite of reducing the patient´s quality of life.

**Methods:**

this was a cross-sectional study aimed to determine the prevalence and factors associated with depression among CKD patients, whereby a practical sampling technique was used among patients attending Bugando Medical Centre, a tertiary level hospital in Mwanza, Tanzania. A total of 376 CKD patients were recruited and interviewed by using patient health questionnaire-9 (PHQ-9).

**Results:**

the mean age of the participants was 52.78 years, ranging from 18 to 85 years, 67.02% were males; 33.24% were more than 60 years of age and 32.45% of the participants were attending hemodialysis at BMC Nephrology Unit. The prevalence of depression was 73.93%. Participants who were 53-96 months since diagnosis, were not affected by modification of lifestyle and who were not sexually active were less likely to develop depression.

**Conclusion:**

the results showed a high prevalence of depression among CKD patients. This emphasizes the need for measures such as earlier screening and management. Which would reduce the burden of morbidity and mortality and improve the quality of life of CKD patients.

## Introduction

According to the World Health Organization (WHO) 2015 global health estimates, 4.4% of the global population were depressed with the prevalence higher in women (5.1%) compared to men (3.6%) [1]. Depression is among the common psychological illnesses among patients with chronic diseases like Chronic Kidney Disease (CKD) [2] and is of poorly studied [3]. Depression is independently associated with increased risk of mortality in CKD patients [4] and multiple studies have proved that depression is more prevalent in CKD patients with end stage renal disease (ESRD) and that it increases the rate of progression from CKD to ESRD [5-7]. Despite dialysis being proven to help in CKD patient´s quality of life, it is a prolonged treatment, which only acts as a bridge to renal transplant [4]. CKD patients are required to attend to the on-going psychological adjustments over the course of their disease such as accepting the diagnosis, medications, frequent hospitalizations, the need for lifelong treatment (dialysis) and its complications [8]. It has also been reported that patients undergoing haemodialysis experience greater symptoms of anxiety and depression as compared to those undergoing peritoneal dialysis [9,10]. Medication adherence, dialysis withdrawal, premature death and suicide are more prominent in CKD patients with major depression and evidence suggests that they die 10 to 20 years earlier than patients without psychological disorders [11].

In Tanzania, studies on the prevalence of depression have been conducted in patients with chronic diseases such as tuberculosis, HIV-AIDS, and women of reproductive age but little is known about patients with CKD [12-14]. Hence the aim of this study was to determine the prevalence and factors associated with depression among CKD patients attending at Bugando Medical Centre, Northwestern Tanzania.

## Methods

**Study design and settings:** this study was conducted at Bugando Medical Centre, a tertiary referral, teaching and research institution for the Lake and Western zones of the United Republic of Tanzania. The hospital has 1,000 beds and serves a catchment population of approximately 15 million people. The Nephrology Clinic and Dialysis Unit attends to 100-130 patients per month [15].

**Sample size, participant´s enrolment, and data collection:** the sample involved both inpatient and outpatients with CKD at Bugando Medical Centre. A minimum sample size of 369 participants was estimated from the Kish-Lisle formula of cross sectional studies, assuming about 40.2% of patients with CKD will have depression [16]. A practical sampling approach from the clinic and the wards was used to recruit the participants from August 2020 to September 2020. Participants were approached at the Nephrology Unit of BMC when receiving hemodialysis. Others were recruited from the CKD clinic day on Mondays, diabetes clinic on Wednesdays, and cardiac clinic on Fridays and also from the BMC wards. Participants were thoroughly briefed about the nature and aim of the study and inclusion and exclusion criteria were applied. Participants who met the inclusion criteria and sign the informed consent were asked to complete two self-administered research questionnaires: a socio demographic questionnaire followed by the patient health questionnaire (PHQ-9). PHQ-9 is a self-reported 9-item scale with empirically demonstrated reliability (Cronbach´s α=0.78) [17]. This scale has been extensively tested and widely accepted for global populations [18]. The PHQ-9 scale has been used and tested in various African countries and translated into many languages including Swahili [17,19]. All recruited participants stayed in the study.

**Data analysis:** data was analyzed using STATA version 13.0 software for Mac. Categorical variables were summarized using frequencies and percentages. Continuous variables were summarized using medians with IQR. Descriptive analysis was conducted to describe the socio-demographic characteristics and the prevalence and severity of depression. Participants, were defined as having depression if they scored above four on the PHQ-9 [20]. Logistic regression was conducted to assess the association between socio-demographic characteristics and depression, controlling for possible confounders. Variables in the univariate analysis that showed a significant effect on the dependent variable were included in the multivariable analysis. Unadjusted and adjusted odds ratio (AOR) with 95% confidence interval (95% CI) were computed and reported where appropriate.

**Ethics:** ethics approval to conduct and publish the findings from this study was given from Catholic University of Health and Allied Sciences/Bugando Medical Centre Joint Ethical Committee with an ethical clearance certificate number CREC/439/2020 and a further permission to conduct this study was granted by Bugando Medical Centre (BMC) administration. Client´s identifiers were not used in analysis to further maintain confidentiality.

## Results

**The socio-demographic characteristics of the study participants:** a total of 376 CKD patients were recruited to participate in the study. The mean age of the participants was 52.78 years, ranging from 18 to 85 years. More than a half of the study participants, 67.02% (n=252), were males with majority of the participants, 65.43% (n=246), permanently residing in an urban area. The majority of the participants, 33.24% (n=125) were more than 60 years of age with more than half of the participants 55.59% (n=209) attaining primary school education. More than two-thirds of the participants, 70.74% (n=266) had been diagnosed with CKD in the past year and only one-third of the participants, 32.45% (n=122), were attending hemodialysis. More than two-third of the participants, 77.93% (n=293), had medical insurance which covers their treatment. Table 1 summarizes the socio-demographic characteristics of the study participants.

**Table 1:** socio-demographic characteristics of CKD patients attending BMC

	Variable	Frequency (n)	Percentage (%)
Gender	Male	252	67.02
	Female	124	32.98
Age (years)	18-30	21	5.59
	31-40	56	14.89
	41-50	94	25.00
	51-60	80	21.28
	>60	125	33.24
Marital status	Married	299	79.52
	Not married	77	20.48
Number of children	0	16	4.26
	1-4	206	54.79
	> 4	154	40.96
Education level	No formal education	35	9.31
	Primary school level	209	55.59
	Secondary school level	104	27.66
	College and above	28	7.45
Income in TZS	≤ 300,000	300	79.79
	300,001-500,000	41	10.90
	500,001-999,999	17	4.52
	1,000,000 and above	18	4.79
What is your permanent resident?	Urban	246	65.43
	Rural	130	34.57
Duration of CKD since diagnosis (months)	2-12	267	70.01
	13-36	85	22.61
	37-52	17	4.52
	53-96	7	1.86
Have you started hemodialysis?	No	254	67.55
	Yes	122	32.45
Do you have Insurance?	No	83	22.07
	Yes	293	77.93
If no insurance who is paying medical expenses?	Family	31	38.75
	Loan	1	1.25
	Relative	15	18.75
	Self	33	41.25
Do you have a treatment supporter?	Yes	336	89.36
	No	40	10.64
Can you perform your activities of daily living?	Yes	214	56.91
	No	162	43.09
What is your current generating income activity?	Unemployed	201	53.46
	Employed	59	15.69
	Self-employed	76	20.21
	Peasants	40	10.64
Can you perform your income-generating activity?	No	238	63.30
	Yes	138	36.70
Has modification of life>	No	276	73.40
	Yes	100	26.60
Are you sexually active?	No	258	68.62
	Yes	188	31.38
TZS: Tanzanian shilling			

**Figure 1:**
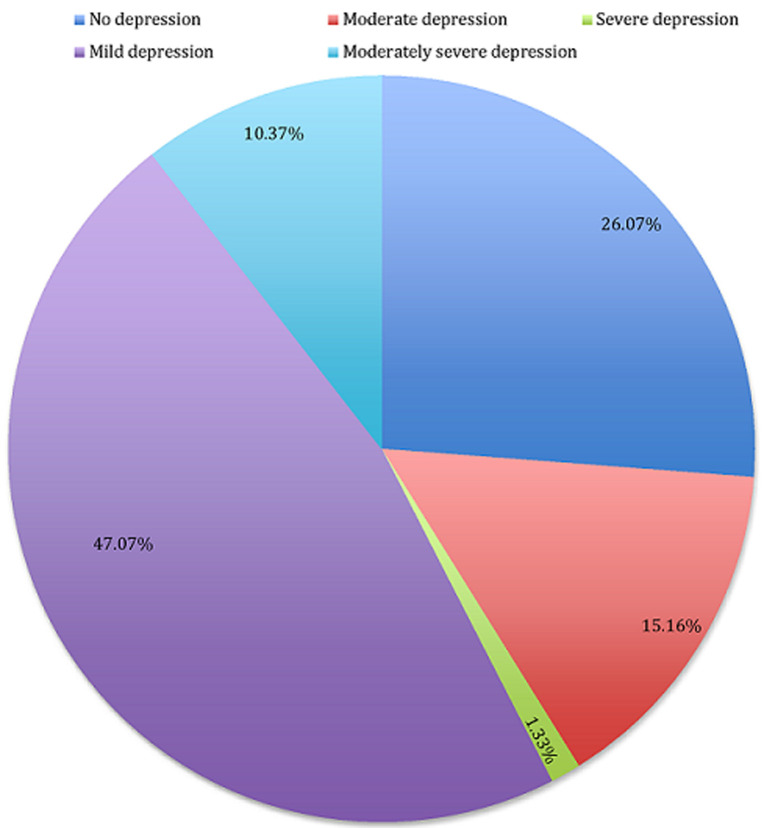
prevalence of depression among CKD patients attending BMC

**Prevalence of depression among CKD patients at BMC:** the prevalence of depression was classified using scores derived from the PHQ-9. Out of the possible maximum score of 27, the lowest score recorded in the sample was 0 and the highest score was 27. Among the 367 participants, 73.93% (n=278) were experiencing depression (PHQ-9 score above 4), while 26.07% (n=98) had no symptoms of depression (PHQ score of 4 and below). As Figure 1 illustrates, 47.07% of the participants had mild depression (PHQ score 5-9), 15.16% had moderate depression (PHQ score 10-14), 10.37% had moderately severe depression (PHQ score 15-19) and 1.33% had severe depression (PHQ score of 20 and above). Figure 1 summarizes the prevalence of depression among CKD patients at BMC.

**Factors associated with depression among CKD patients:** in the unadjusted model, duration since diagnosis, ability to perform activities of daily living, ability to perform income-generating activities, modification of lifestyle and sexual activity were significantly associated with depression. After adjusting for other factors associated with depression, those who could not perform activities of daily living (ADLs) were significantly less likely to have depressive symptoms (AOR 0.32, 95% CI: 0.17,0.61, P-value=0.001) as compared to those who could perform them.

In univariate analysis, participants being 53-96 months since diagnosis were less likely to develop depressive symptoms (AOR 0.56, 95% CI: 0.32, 0.99, P-value=0.044) as compared to those with 3-12 months after diagnosis. While in multivariate analysis they were not significant. Those who were not affected by modification of lifestyle were significantly less likely to develop depression symptoms in univariate analysis (AOR: 0.54, 95% CI: 0.33,0.90, P-value=0.018) while in multivariate there were no significant association. In univariate analysis participants who were not sexually active were significantly less likely to develop symptoms of depression (AOR 0.47, 95% CI: 0. 29, 0.76, P-value =0.002) while in multivariate analysis they were not significantly associated as compared to those who were sexually active. Table 2 summarizes the association between sociodemographic factors and depression.

**Table 2:** association between socio-demographic factors and depression among CKD patients attending BMC

Variable	Depression		Univariate		Multivariate	
	No (N%)	Yes (N %)	UOR (95%CI)	P-value	AOR (95%CI)	P-value
**Gender**						
Female	27 (21.77)	97 (78.23)	1.0			
Male	71 (28.17)	181 (71.83)	0.70 (0.42-1.17)	0.185	-	-
Age (years)						
18-30	5 (23.81)	16 (76.19)	1.0			
31-40	13 (23.21)	43 (76.79)	1.03(0.31-3.36)	0.956	-	-
41-50	20 (21.28)	74 (78.72)	1.15(0.37-3.54)	0.799	-	-
51-60	24 (30)	56 (70)	0.72 (0.23-2.21)	0.578	-	-
>60	36 (28.80)	89 (71.20)	0.77 (0.26-2.26)	0.638	-	-
**Marital status**						
Married	77(25.75)	222 (74.25)	1.0			
Not married	21(27.27)	56(72.73)	1.08(0.61-1.90)	0.786	-	-
**Education**						
I have no formal education	10 (35.71)	18 (64.29)	1.0			
Primary school level	50 (23.92)	159(76.08)	1.2(0.5-2.8)	0.55	-	-
Secondary school level	28 (26.92)	76 (73.08)	1.1 (0.4-2.5)	0.850	-	-
College and above	10 (35.71)	18 (64.29)	0.7 (0.2-2.1)	0.546	-	-
**Time since diagnosis**						
2-12 months	58(21.72)	209(78.28)	1.0			
13-36 months	31(36.47)	54(63.53)	0.48(0.28-0.82)	0.007	0.56(0.18-1.75)	0.317
37-52 months	5(29.41)	12(70.59)	0.66(0.22-1.97)	0.462	0.29(0.06-1.44)	0.131
53-96 months	4(57.14)	3(42.86)	0.20(0.045-0.96)	0.044	0.56(0.32-0.99)	0.044
**Have you started dialysis?**						
Yes	30(24.59)	92(75.41)	1.0			
No	68 (26.77)	186 (73.23)	1.121(0.68-1.84)	0.652	-	-
**Can you still perform your activities of daily living?**						
Yes	20(20.41)	78 (79.59)	1.0		1.0	
No	142 (51.08)	136 (48.92	0.24 (0.14-0.42)	< 0.001	0.32(0.17-0.61)	<0.001
**Can you still perform your income generating activities?**						
Yes	51 (36.96)	87 (63.04)	1.0		1.0	
No	47 (19.75)	191 (80.25)	0.41(0.26-0.67)	0.000	0.81(0.44-1.50)	0.499
**Has modification of life>**						
Yes	35 (35.00)	65 (65.00)	1.0		1.0	
No	63 (22.83)	213 (77.17)	0.54(-.33-0.90)	0.018	0.73(0.42-1.26)	0.264
**Are you sexually active?**						
Yes	43 (36.44)	75 (63.56)	1.0		1.0	
No	55 (21.32)	203 (78.68)	0.47 (0.29-0.76)	0.002	0.76(0.42-1.40)	0.38

## Discussion

The health burden results from the rapid shift from communicable diseases to non-communicable diseases (NCDs), and with the shift the health systems for low- and middle-income countries (LMICs) such as Tanzania were unprepared for the change with little access to preventive and primary care resulting in the high mortality and morbidity rate [21] leading to further exacerbation of poverty [22]. From this study more than a half of the participants were male, probably due to males being at more risk of developing CKD as they have a higher incidence of smoking, alcohol abuse and engage less in health seeking behavior [23]. This observation was also found in other studies conducted in Brazil [24] and China [25]. Chronic kidney disease continues to be a disease of people with advanced age, as was evidenced in this study, with the majority of the participants 60 years and over which is similar to Chinese [26], Russian [27] and Korean [28]. Epidemiological evidence suggests that vascular diseases including diabetes, hypertension and obesity may be the leading etiology for CKD in this population since they are prevalent in CKD patients and are associated with albuminuria and decreased glomerular filtration rate (GFR) [29]. Older people are also at risk of social isolation and depression [30].

The majority of the participants had a primary school education level which aligns with other studies conducted in Ethiopia [31] and Taiwan [32]. Those with lower socioeconomic profiles defined by education have been reported in previous studies to face problems in their psychological wellbeing and general health [33,34]. With regard to marital status, more than half of this study´s participants were married which corresponds with a study conducted in Nigeria [35]. This is because CKD mostly affects older people who are more often married if they are over the age of 40 years [36]. The results are also in contrast with studies conducted in China [37] and Ethiopia [31] where more than half of the participants were not married. This could be due to cultural differences, religion, being widowed and the high rate of divorce [26,38].

The overall prevalence of depression in this study was 73.93% which is in contrast with other studies. An Ethiopian study had a prevalence of 29.4% [31]; a Nigerian study had a prevalence of 34.8% [27]; a Saudi Arabian study had prevalence of 24.6% whilst a Singaporean study had a prevalence of 49.9% [28]. The difference in results is probably due to a difference in sample size and different methodologies. This study used the PHQ-9 whilst other studies used the Hospital Anxiety and Depression scale (HADs) and Beck Depression Inventory (BDI). Cultural differences also play a big role in depressive symptoms. Culture as a way of life of a group of people plays a significant role as it is composed of beliefs, values, norms and behaviors which are passed from generation to generation and change over time [39]. The response of African CKD patients is certainly different those in Europe or the USA since culture influences how individuals manifest and communicate symptoms, cope with their psychological challenges and their readiness to seek medical treatment [39].

In this study the majority of the participants were not sexually active and were 0.47 less likely to have depression as compared to participants who were sexually active. This is similar to the Greek study [40] which related sexual performance significantly with a negative association among CKD patients. This is as a result of multiple factors such as hormonal disturbances, medications including antihypertensives and antidepressants and comorbid diseases such as diabetes, cardiovascular diseases and malnutrition [41-43]. Modification of lifestyle is associated with depression in CKD patients whereas in this study participants who were not affected by modification of lifestyle were significantly less likely to develop depressive symptoms, which is also similar to the study conducted in USA among Veterans Affairs Medical Centre [44].

Depression was also associated with the participants´ ability to perform their income generating activities whereby participants who could not perform their income generating activities were significantly less likely to develop depressive symptoms compared to those who could perform their income generating activities. The results are also in contrast to a study conducted in China whereby a Kanrosfky performance status was conducted and found out that patients who could not perform their duties having a score >90 were having more depressive symptoms [45]. This is because depression has a serious impact on cognitive, social and physical function and is related to work impairment, disability, lost work days and reduced productivity at work [46]. This also affects the participant as it becomes hard to find and keep and maintain a job [47]. Diagnosis of the disease was also a risk factor and in this study participants having been diagnosed with CKD within 4 years and above (53-96 months) were 0.2 times less likely of having depression which is similar to studies conducted in China [45] and India [48] whereby participants with depressive symptoms having more than 4 years durations since diagnosis of CKD had improved quality of life [49].

In order to manage and maintain psychosocial health of CKD patients, the study recommends earlier screening/detection of disorders, increasing the number of centers providing dialysis services in hospitals within the country and appropriate intervention to manage psychosocial illness such as psychiatrists and counselors. Future studies should include understanding the quality of life among CKD patients, post-traumatic stress disorder among CKD patients, prevalence of depression among CKD caregivers and post-transplant patients, treatment outcome studies to detect the most effective combinations of treatment strategies for depression and other mental disorders and examining the effectiveness of intervention strategies for mental disorders. The main limitation of this study was its cross-sectional nature whereby causality could not be assessed between factors examined. Therefore, there is need for longitudinal studies to establish the exact causal relationship between depression and the investigated variables.

**Limitations:** a cross-sectional study was used which relies on self-report of symptoms which could lead to recall bias. Non-generalizability of the findings is another limitation, despite that the study was done at the largest hospital in the lake and western zone of Tanzania which serves a diverse population still regional difference could be there.

## Conclusion

In this study, depressive symptoms were high among CKD patients, which calls for attention to mental health prevention, care, and treatment. Continued advocacy about mental health may help create awareness of primary preventive interventions including anti stigma campaigns such as mental health awareness week with increases in mental health services including screening, diagnosis and management among CKD patients which is crucial for secondary prevention.

### What is known about this topic

- Depression is among the common psychological illnesses among patients with chronic diseases like chronic kidney disease (CKD) but poorly studied in LMICs;
- Depression is more prevalent in CKD patients with inability to perform activities of daily living (ADLs), who are sexually inactive and those with lifestyle modification.

### What this study adds

- A high prevalence of 73.93% was reported in this study from a middle-income country;
- Surprisingly inability to perform ADLs, sexually inactive or life style modification did not increase the likelihood of having depression among CKD patients in this study.
